# Posterior mediastinal hematoma – a rare case following a fall from standing height: a case report

**DOI:** 10.1186/1752-1947-1-185

**Published:** 2007-12-28

**Authors:** Lakshmi Pasumarthy

**Affiliations:** 1 Department of Medicine, York Hospital, York, PA, USA; 2 Department of Medicine, Penn State Hershey Medical Centre, Hershey, PA, USA

## Abstract

**Introduction:**

We present a previously unreported complication of a fall from standing height.

**Case presentation:**

A 76-year-old woman sustained blunt chest trauma resulting from a fall from standing height. She was diagnosed with a mediastinal hematoma, and did well with supportive care. Follow up CT angiograms on days 2 and 4 of hospital stay revealed a stable hematoma and she did not require any intervention.

**Conclusion:**

Mediastinal hematoma has been reported secondary to trauma, coagulation abnormalities and hematologic malignancies, but it not been reported secondary to a fall from standing height. Factors predisposing to a hematoma in this case were aspirin therapy and a modest elevation of INR secondary to chronic hepatitis C.

## Introduction

Falls from a standing height are common in the elderly. The patients usually present with pain due to fractures or soft tissue injury. Occasionally more severe complications such as sub-dural hematoma may result from the trauma, but mediastinal hematoma has not been reported.

## Case presentation

A 76 year-old woman presented after falling in the bathroom. She had become dizzy and her face struck the edge of the sink as she fell. She landed on bilateral outstretched arms. She complained of severe pain in her shoulders and was taken to the emergency department. She received intravenous fentanyl. She became nauseated and vomited. Soon after that she complained of shortness of breath. On arrival at the emergency room, her room air oxygen saturation was 98% but later, during the episode of respiratory distress, it dropped to 85%. BP on presentation was 134/71, pulse rate was 74.

Two weeks prior to the fall, a CT scan of the chest had been performed to evaluate persistent cough. The CT scan at that time did not reveal any masses or aneurysms. Past medical history was significant with a history of hepatitis C diagnosed in 2004, confirmed by serologic testing and biopsy. She also had acid reflux, coronary artery disease, requiring a stent to the left anterior descending artery in 2004, severe degenerative joint disease, and lumbar spine surgery in 1991.

She was being treated with aspirin 81 mg (for coronary artery disease), spironolactone (for early cirrhosis), furosemide (for early cirrhosis), omeprazole (for acid reflux), and gabapentin (for chronic neck and back pain).

Social history was noteworthy for lack of alcohol use and smoking. She lived with her husband of 56 years.

On initial examination, she was in pain, but the rest of the examination was within normal limits, including auscultation of heart and lungs. After the episode of respiratory distress, she was intubated, and bruising was noted over the left eyelid and neck. Her pupils were equal and round to light and accommodation. Examination of the neck did not reveal any jugular venous distention or carotid bruit. Auscultation of the lungs revealed inspiratory stridor, heart sounds were heard well with no murmurs, rubs or gallops. Her chest wall was without any obvious hematoma or deformity. Her abdomen was not distended and no hepatosplenomegaly was detected.

**Figure 1:**
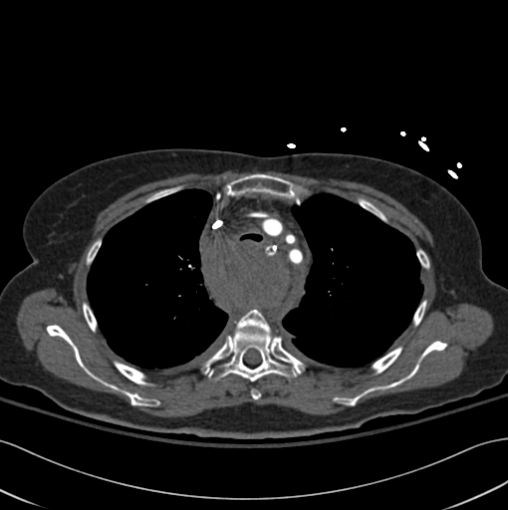
Posterior Mediastinal hematoma-high mediastinum – Please note the compressed trachea.

EKG showed normal axis, poor R wave progression. X-rays did not reveal any fractures, dislocations of the shoulder joint or vertebral fractures. Chest X-ray showed a widened mediastinum. CT scan of the chest was performed to better assess the widened mediastinum and revealed a large posterior mediastinal hematoma. (Figure 1). There was no active bleeding noted from any of the major vessels.

In the emergency room, platelet count was 95,000/cu mm, INR was 1.3, and hemoglobin was 10.6/cu mm. Sodium was 118 mmol/L and the rest of her renal panel was normal. Her prior sodium levels were between 120 and 125 mmol/L, felt to be secondary to diuretic therapy. Potassium was 5.2 mmol/L, chloride was 87 mmol/L, liver chemistries revealed albumin of 2.42 g/dl, alkaline phosphatase of 185 U/L, aspartate aminotransferase 110 U/L, alanine aminotranferase of 62 U/L, calcium was 8.3 mg/dl, and magnesium was 1.5 mg/dl. She had been negative for cryoglobulins and anti nuclear antibodies. On comparing the platelet count, INR and liver chemistries with recent blood work no changes were found, and the findings were felt to be the result of the cirrhosis.

CT scan of the chest revealed findings as above. 2-D Echocardiogram revealed normal ventricular size with hyperdynamic function, moderate mitral regurgitation, and moderate tricuspid regurgitation.

Follow-up CT scans on days 2 and 4 revealed no progression of the hematoma. She continued not to require vasopressor support while in the ICU. On the fourth day she extubated herself, and did not develop respiratory distress. She was transferred to a general ward, observed for several days and later transferred to a rehabilitation center.

## Discussion

Blunt trauma to the chest wall occasionally results in bleeding within the mediastinum, such as seen in motor vehicle crashes or fall from heights of 6 meters or greater [1,2]. The mechanism is felt to be secondary to rapid deceleration and luminal pressure against points of fixation (ligamentum arteriosum). Anecdotal reports of coagulation abnormalities and neoplasms causing mediastinal hematomas have also been published. [3,4]

Sources of posterior mediastinal hematoma are rupture of the descending aorta, ruptured aneurysm of the inferior thyroid artery, and vertebral fractures. Sources of anterior mediastinal hematoma include rupture of an internal mammary artery, and sometimes due to hemorrhage from thyroid gland or thymus. In many patients who survive the acute episode it is felt that the source of bleeding is from smaller arteries and veins.

Anterior mediastinal masses can be identified when the hilum overlay sign is present and the posterior mediastinal lines are preserved. If the bifurcation of the main pulmonary artery is >1 cm medial to the lateral border of the cardiac silhouette, it is strongly suggestive of a mediastinal mass. An anterior mediastinal mass that appears as an enlarged cardiac silhouette will not cause displacement of the pulmonary arteries. A mediastinal mass that projects superior to the level of the clavicles must be located either within the middle or posterior mediastinum.

Although aortography has long been considered the gold standard for the diagnosis of traumatic aortic rupture, contrast-enhanced spiral computed tomography of the chest constitutes an accurate alternative imaging modality. Trans-esophageal echocardiogram (TEE) appears to be an accurate method to diagnose traumatic mediastinal hematoma. [5] Chest radiography is the initial screening examination, and radiographs are evaluated specifically for signs of mediastinal hematoma, an indication of significant thoracic trauma. The most important of these signs include loss of aortic contour, tracheal deviation, ratio of mediastinal width to chest width, deviation of a nasogastric tube (when used) to the right of the T-4 spinous process, and depression of the left main-stem bronchus (> 40 degrees below the horizontal). CT is used increasingly when results of chest radiography are equivocal. CT can clearly demonstrate mediastinal hematoma, but this finding is also mimicked by several entities, including atelectatic lung, thymus, and pericardial recesses. [6] Chest X-ray alone is inadequate as a diagnostic tool, since approximately 50% of cases had a normal size mediastinum. [7]

Initial treatment consists of fluid resuscitation, and transfusion as necessary. Endotracheal intubation should be considered if there is a concern about airway compromise. Cardiopulmonary bypass is used if there is evidence of heart failure and short acting beta blockers are recommended to reduce mean arterial pressure to 60 mm Hg and to control heart rate. Endovascular stents are being used more, but further data is necessary. Surgical repair is the definitive treatment especially if there is evidence of ongoing blood loss but delaying this until the patient is more stable lowers mortality rates. [8]

Literature review of case reports, done through Pub Med from 1980 to the present, yielded no previous cases of mediastinal hematoma secondary to a fall from a standing height. 15 cases were secondary to spontaneous hematoma, 19 were reported secondary to complication of subclavian venous cannulation, and 9 were secondary to blunt trauma. The most common presenting complaint in all these cases was shortness of breath. CT scan provided the diagnosis in 8 out of the 9 cases in the blunt trauma category. Echocardiogram was used in one case where cardiac tamponade was suspected and confirmed. 8 of the patients underwent surgery and did well, one died secondary to hemorrhage.

Though our patient's platelet count and INR were not normal, they were unlikely to explain the bleeding in the mediastinum alone. It is possible that aspirin therapy and the predisposition to bleeding due to mild thrombocytopenia and elevated INR may have contributed together. She had no structural abnormality like an aortic aneurysm as evidenced by a normal CT of the chest two weeks prior to the event. There was no evidence of cryoglobulinemic vasculitis. Her hematoma was contained and showed no sign of progression on further testing, and she was hemodynamically stable throughout her hospital course.

## Conclusion

Falls from standing height are common in the elderly, but there are no other cases in the literature describing a resulting posterior mediastinal hematoma of this severity, especially without a background of bleeding diathesis. When symptoms such as sudden onset of chest pain and shortness of breath are present, especially if associated with mediastinal widening on chest X-ray, then mediastinal hematoma should be considered even if the patient has fallen only from a standing height, and has not necessarily struck the chest.

This case exemplifies a rare complication of a commonly encountered issue.

## Abbreviations

CT – Computerized tomography

INR – International normalized ratio

EKG – Electrocardiogram

BUN – Blood urea nitrogen

ICU – Intensive care unit

TEE – Trans-esophageal echocardiogram

## Competing interests

The author(s) declare that they have no competing interests.

## Consent

Written informed consent was obtained from the patient for publication of this case report and any accompanying images. A copy of the written consent is available for review by the Editor-in-Chief of this journal.
